# Supplementary material for: CXCR5 CAR-T cells simultaneously target B cell non-Hodgkin’s lymphoma and tumor-supportive follicular T helper cells
Source: Nat Commun. 2021 Jan 11;12:240. doi: 10.1038/s41467-020-20488-3 (PMC7801647; doi:10.1038/s41467-020-20488-3)
Supplement: Supplementary file 1 — Supplementary Information [file 41467_2020_20488_MOESM1_ESM.pdf]

Supplementary Information

CXCR5 CAR-T cells simultaneously target B cell Non-Hodgkin's lymphoma and tumor-supportive follicular T helper cells

Supplementary Figure 1

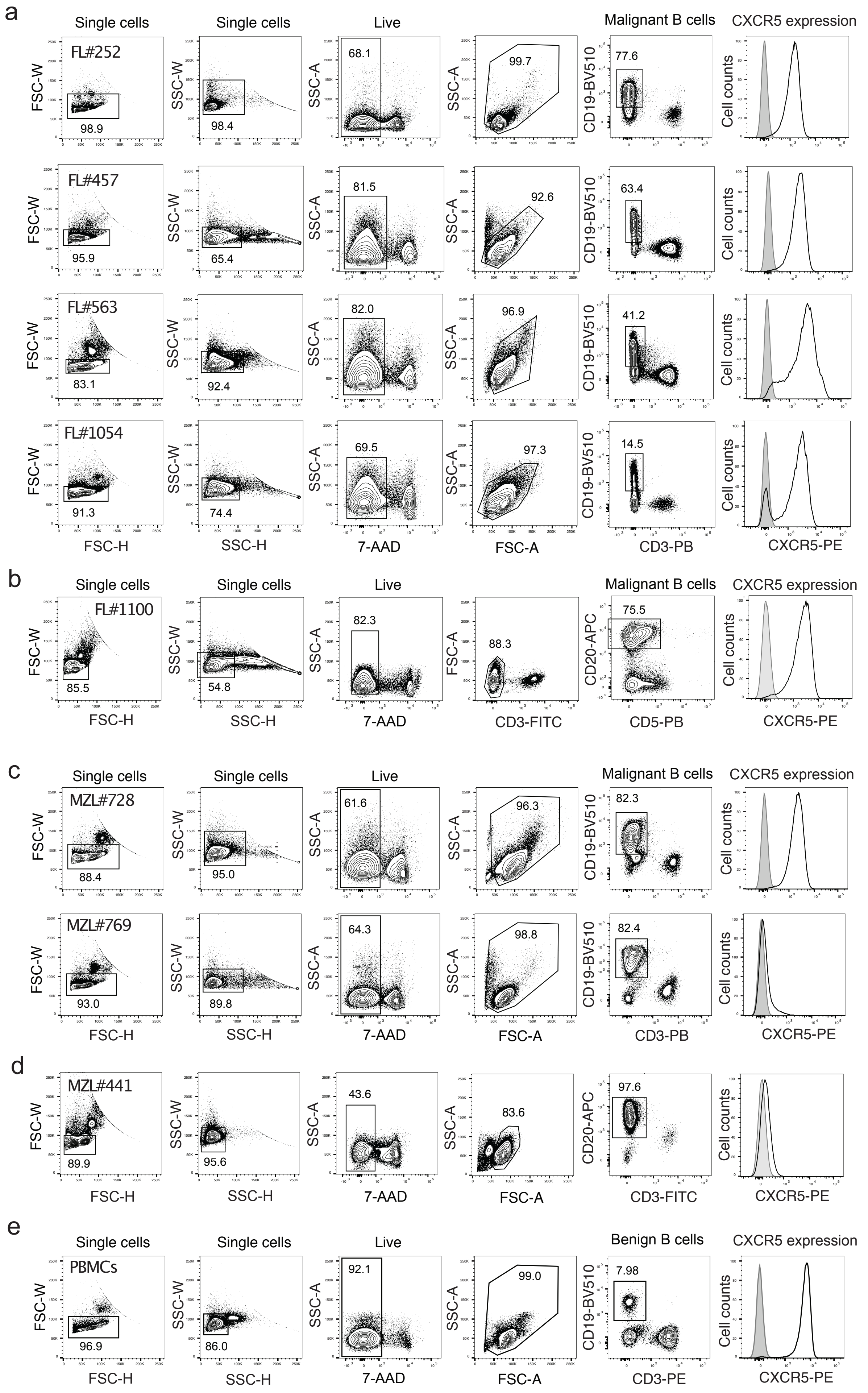

Supplementary Figure 1. Flow cytometry analysis of CXCR5-expressing primary B-NHLs. Primary FL and MZL samples were analyzed with either of two gating strategies: (**a**) Gating strategy based on CD19 and CD3, applied for FL #252, #457, #5632 and #1054. (**b**) Representative example of the gating strategy based on CD20 and CD3 that was applied for FL #85, #1100, and #1129. (**c**) Gating strategy based on CD19 and CD3 that was applied for MZL #728 and #769. (**d**) Representative example of the gating strategy based on CD20 and CD3 that was used for MZL #421 and #441. (**e**) Representative example of the gating strategy based on CD19 and CD3 that was used for benign peripheral blood B cells for comparison.

Supplementary Figure 2

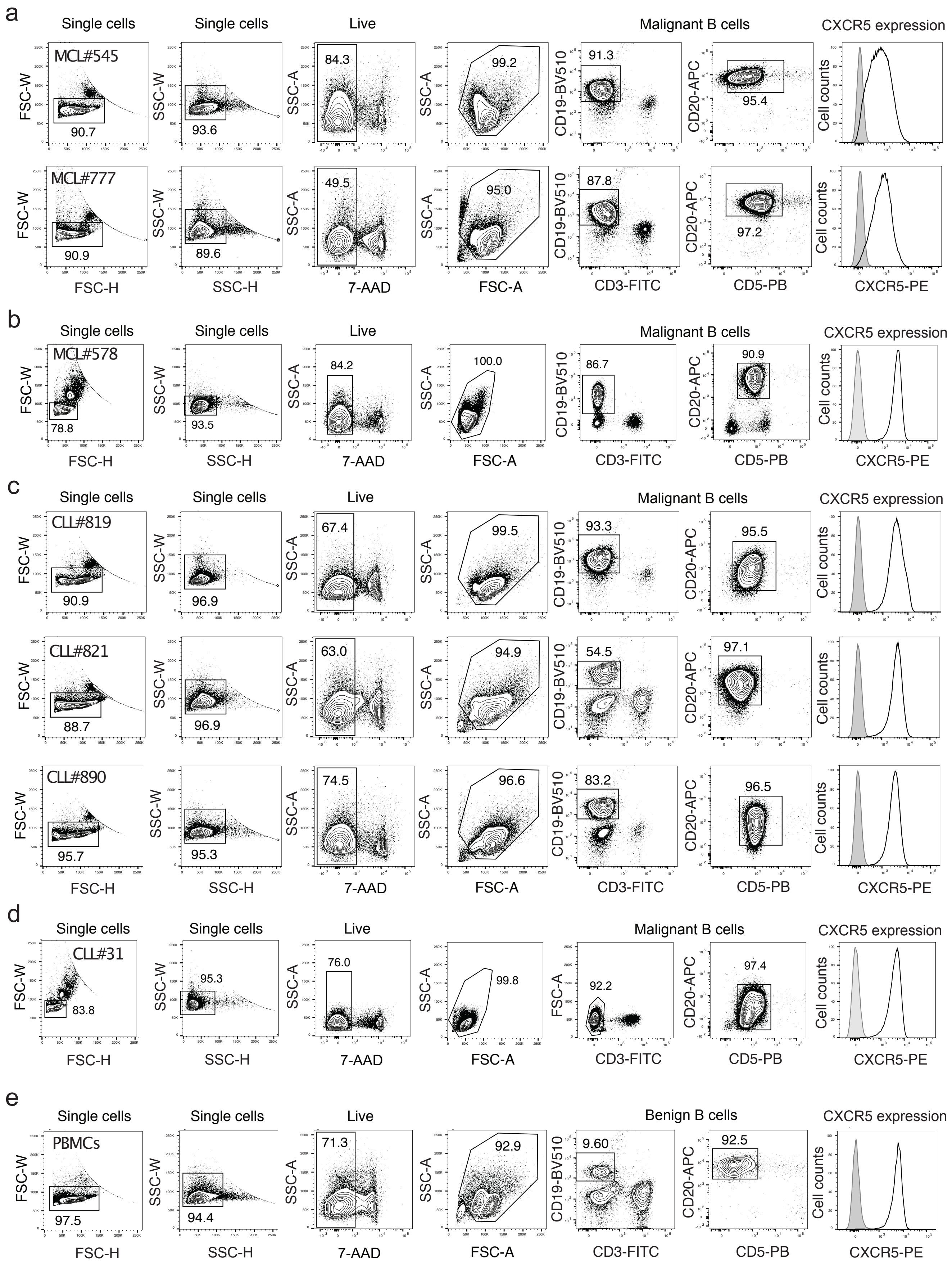

Supplementary Figure 2. Flow cytometry analysis of CXCR5-expressing primary B-NHLs.
(**a**) Gating strategy based on CD3, CD19 and CD20 for primary MCL #545 and #777. (**b**) Representative example of the gating strategy based on CD3, CD19 and CD20 that was used for MCL #114, #578, #624, and #988. (**c**) Gating strategy based on CD3, CD19 and CD20 for primary CLL #819, #821, and #890. (**d**) Representative example of the gating strategy based on CD3, CD20 and CD5 that was used for CLL #12, #31, #33, #47 and #75. (**e**) Representative example of the gating strategy based on CD3, CD19 and CD20 that was used for benign peripheral blood B cells for comparison.

Supplementary Figure 3

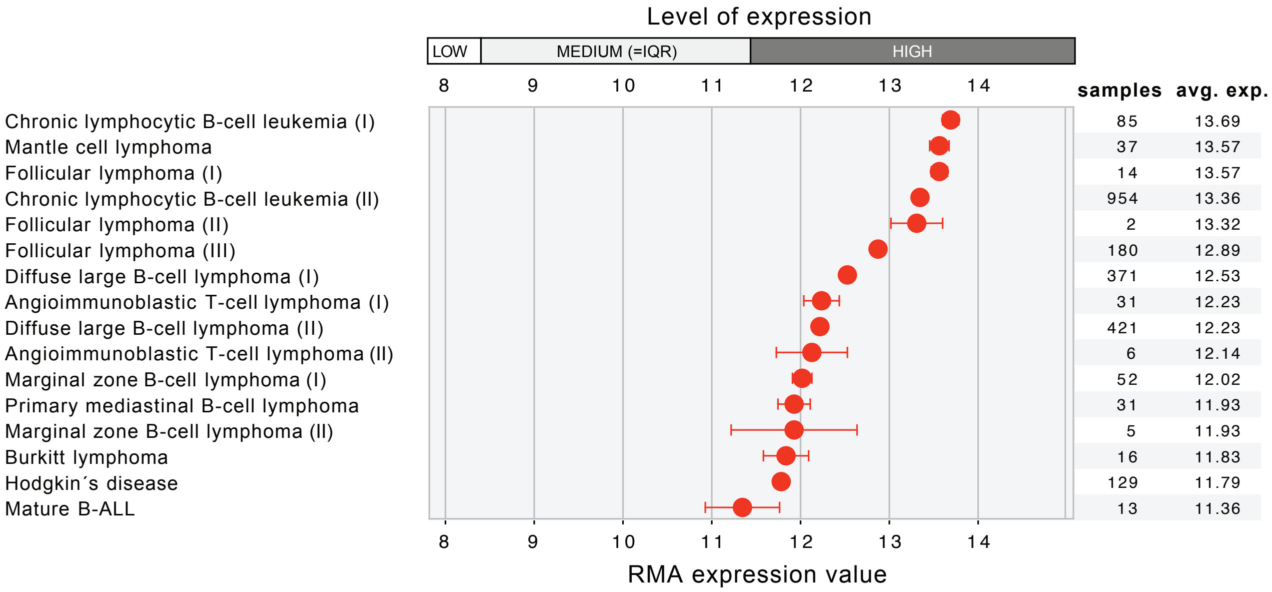

Supplementary Figure 3. Cancer types with the highest CXCR5 expression levels in the genevestigator database.

Shown are the top 16 hits of a query for CXCR5 expression on a curated microarray database (HS_AFFY_U133PLUS_2-0) with 630 cancer categories. The database collects published microarray studies and was interrogated using the Genevestigator V7.4.1 (Nebion) software (www.genevestigator.com, accessed on November 4, 2019). Roman numbers in brackets indicate that cancer types differed in further clinical descriptions. Dots show mean RMA expression values ±SEM.

Supplementary Figure 4

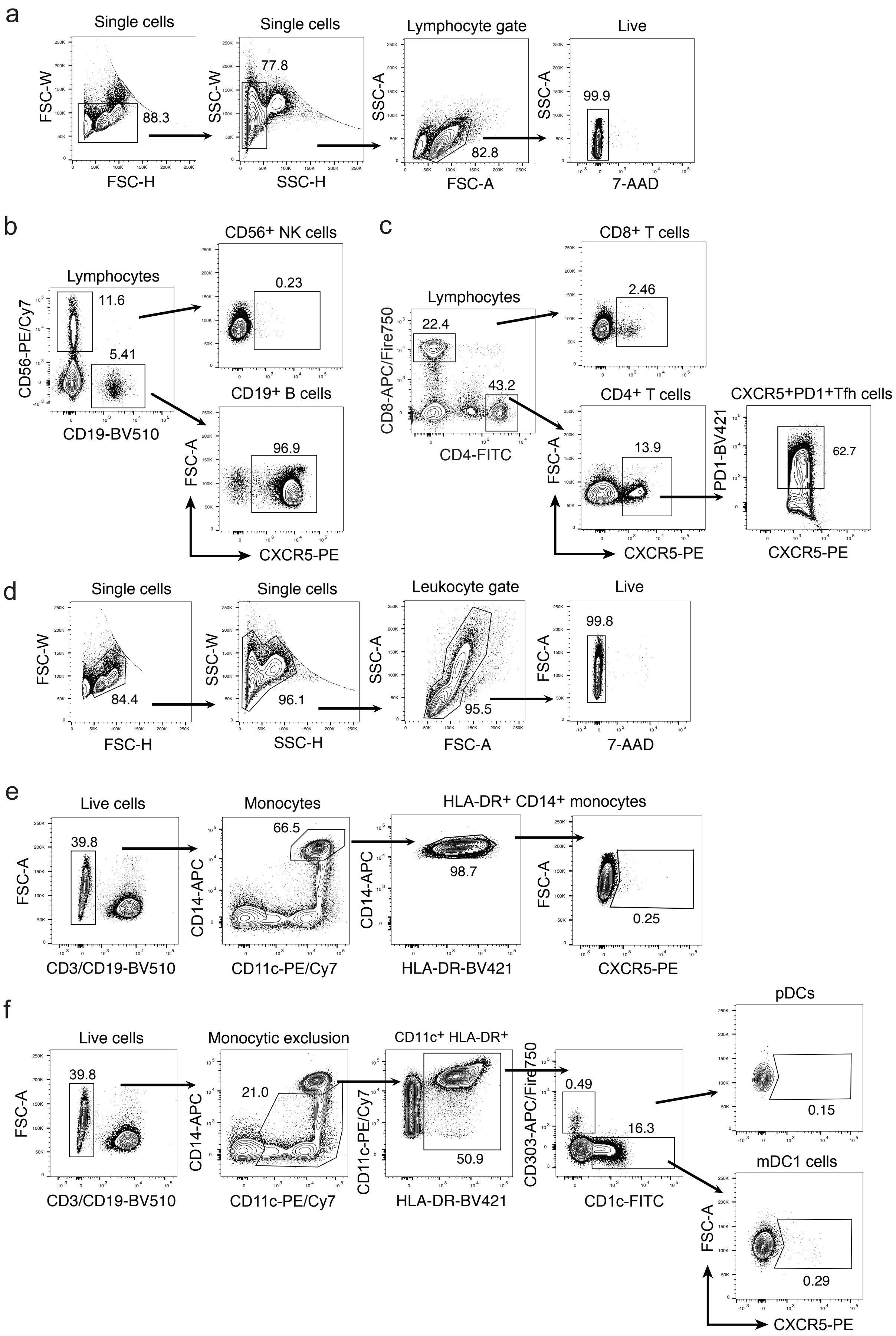

Supplementary Figure 4. CXCR5 is not expressed on the majority of peripheral blood T cells, NK cells, and myeloid cell subsets.

Pre-gating strategy for live (**a**) lymphocytes and (**d**) all leukocytes from PBMCs, isolated by density gradient centrifugation from peripheral blood of healthy donors. Samples were analyzed by flow cytometry for the percentage of CXCR5 expression on $CD56^{+}$ NK cells and $CD19^{+}$ B cells (**b**), $CD4^{+}$ and $CD8^{+}$ T cells, $CD4^{+}PD1^{+}$ Thf cells (**c**); monocytes (**e**), and pDC and mDC1 cells (**f**). Monocytes were identified by gating on $CD3^{-}/CD19^{-}/CD14^{+}/CD11c^{+}/HLA\text{-}DR^{+}$ cells. DCs were identified by gating on $CD3^{-}/CD19^{-}/CD14^{-}/CD11c^{+}/HLA\text{-}DR^{+}$ cells and then on $CD303^{+}/CD1c^{-}$ (pDC), or $CD303^{-}/CD1c^{+}$ (mDC1). Numbers in the plots indicate percentages of the gated population, respectively. Plots show representative data of a single donor out of n=5 donors investigated for each leukocyte subpopulation.

Supplementary Figure 5

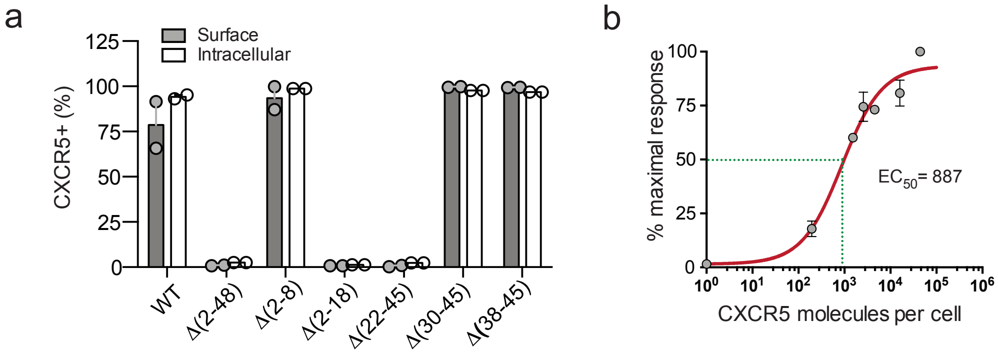

Supplementary Figure 5. Binding epitope and affinity of the anti-human CXCR5 mAb.
(**a**) The scFv sequence of the anti-CXCR5 CAR is based on the rat anti-human CXCR5 mAb RF8B2. The RF8B2 epitope was mapped using Jurkat cell lines expressing CXCR5 deletion variants. Deletions were located in the extracellular N-terminus (1-55) as indicated. CXCR5-negative Jurkat cells were transduced with the CXCR5 variants and GFP as a transduction marker, stained with the RF8B2 mAb for surface and intracellular protein, and analyzed by flow cytometry. The graph shows the percentage of GFP-positive, transduced cells that were stained by the mAb. Jurkat cells expressing the unmodified wildtype CXCR5 (wt) served as positive control. Bars represent means ±SEM and the graph shows data of n=2 independent experiments. **(b**) The affinity of CXCR5 CAR-T cells was determined using clonal Jurkat cell lines expressing graded amounts of CXCR5. The amount of surface CXCR5 per cell was quantified using the QuantiBRITE^{TM} bead method. CXCR5 CAR-T cells were co-cultured with selected cell lines and afterwards IFN in the supernatant was quantified by ELISA. The amount of CXCR5 molecules per cell that elicited the half maximal cytokine secretion (EC50) was calculated using a nonlinear regression curve (log [agonist] vs. response; three parameters) using Prism, version 6.0. The graph shows means ±SEM of n=3 donors. Source data are provided as a Source Data file.

Supplementary Figure 6

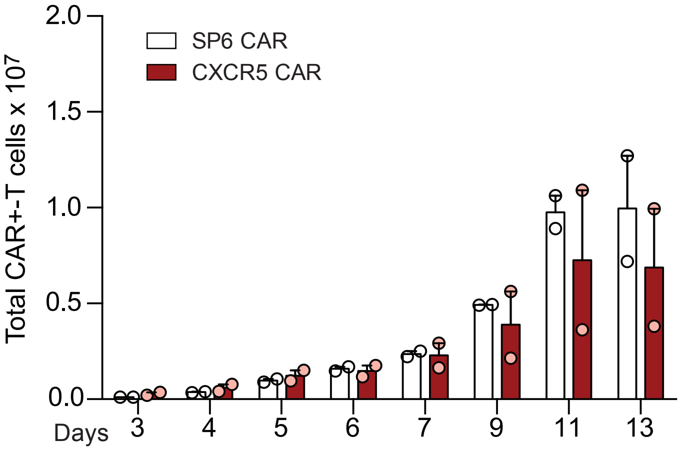

Supplementary Figure 6. Minor reduction of CXCR5 CAR-T cell expansion in small-scale cell culture.

Primary T cells were seeded, activated with TransAct, transduced with either SP6 CAR or CXCR5 CAR in the presence of Vectofusin-1 and grown in TexMACS medium supplemented with IL7 and IL15 (both 10ng/mL) and until day 5 with 1% human AB-serum. The numbers of CAR^{+}-T cells were counted from day 3 to 7 daily and thereafter every other day. To account for the loss of cells that were used for counting, the expansion rate was calculated and the hypothetical number of proliferated cells was added to the actual cell count. Bars represent means ±SEM of n=2 donors. Source data are provided as a Source Data file.

Supplementary Figure 7

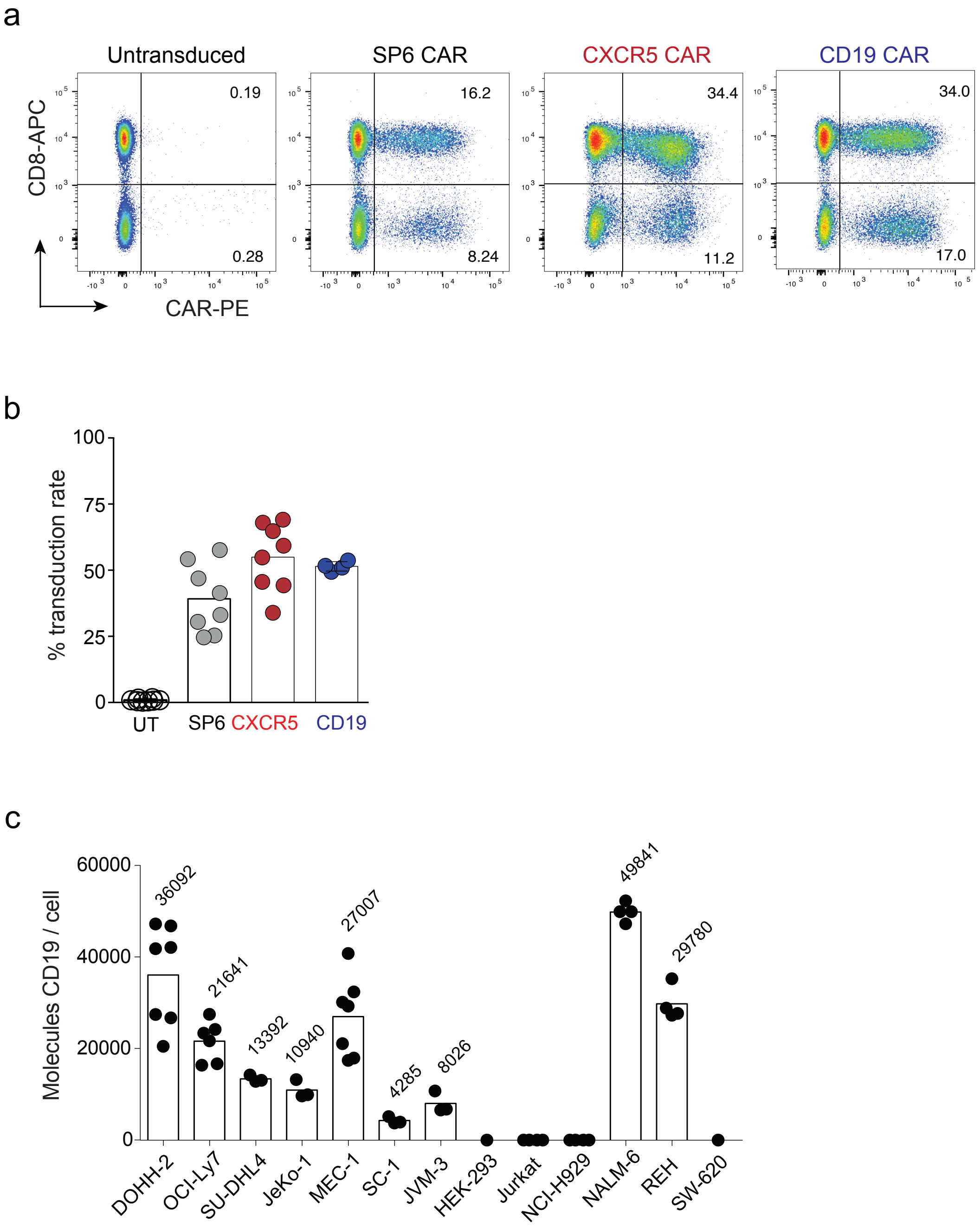

Supplementary Figure 7. Transduction rates are similar for CD19 and CXCR5 CARs and CD19 is highly expressed on B-NHL, B-CLL and B-ALL cell lines.

(**a**) CAR surface expression on human T cells transduced with CD19, CXCR5 and SP6 (control) CARs, or left untransduced (UT), analyzed on day 11 after transduction by anti-IgG staining and flow cytometry. Percentages of $CD8^{+}CAR^{+}$ and $CD8^{-}CAR^{+}$-T cells are indicated. (**b**) Bars represent means of the percentage of CD19, CXCR5 and SP6 CAR-expressing T cells. The data for UT, CXCR5 and SP6 CAR-T cells were also used in Fig. 2C and are shown here for comparison. Representative data in (**a**) and in (**b**) were generated by n=4-6 independent experiments and n=4-8 individual donors. (**c**) Quantification of CD19 molecules per cell on B-NHL (DOHH-2 and SC-1, FL; SU-DHL4 and OCI-Ly7, DLBCL; JeKo-1, MCL), on B-CLL (MEC-1 and JVM-3), on MM (NCI-H929), and on T-ALL (Jurkat) cell lines. The kidney epithelial cell line HEK293 and the colorectal adenocarcinoma cell line SW-620 served as negative controls. Data of n=1-7 independent measurements for each cell line is shown. Numbers indicate the mean. Source data are provided as a Source Data file.

Supplementary Figure 8

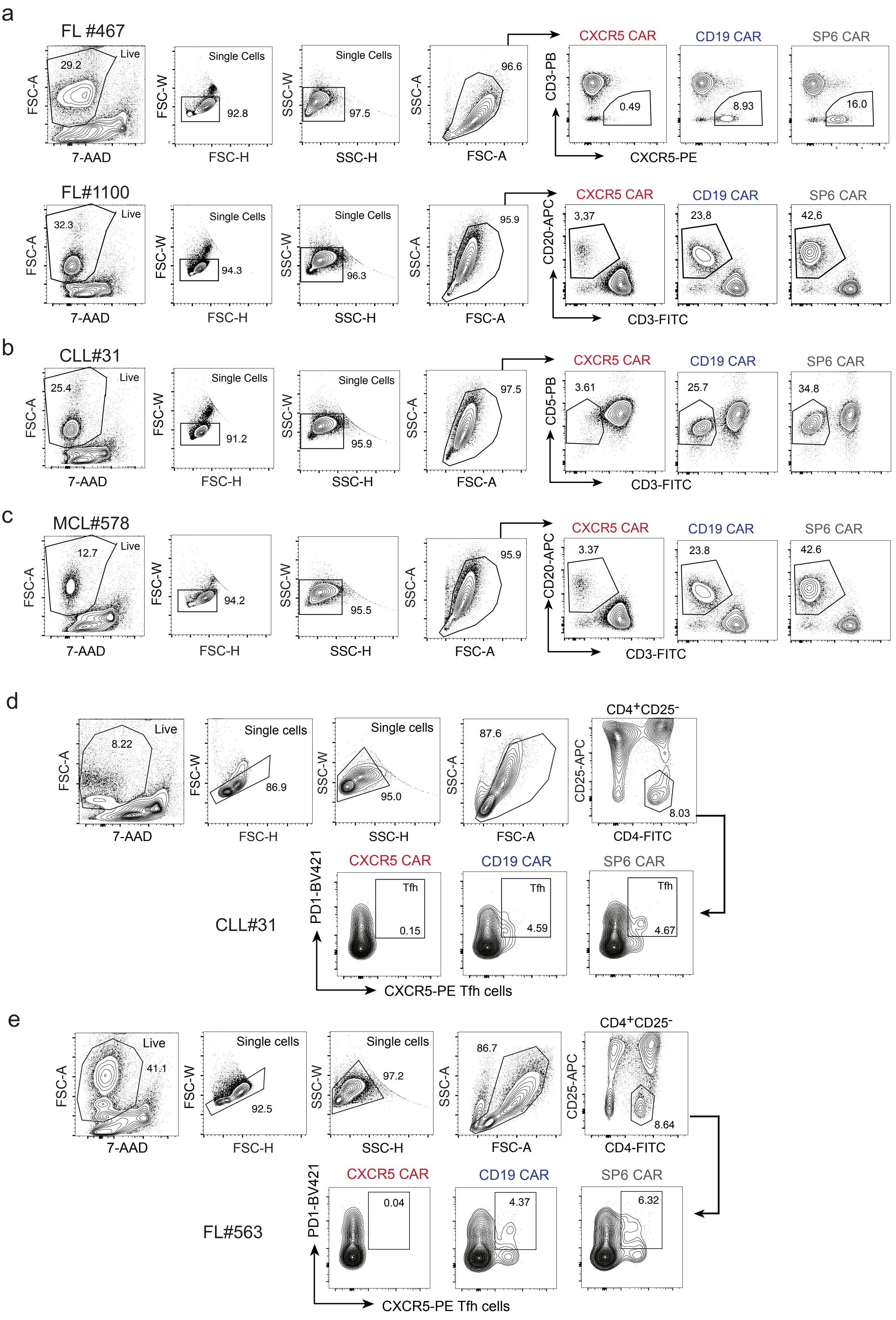

Supplementary Figure 8. Analysis of co-cultures of CAR-T cells and primary tumor samples by flow cytometry.

The percentage of tumor cells and T cells was analyzed using either of two gating strategies. (**a**) The co-cultures of FL #252, #467, #5632 and #1054, and the corresponding JeKo-1 controls were analyzed with a gating strategy based on CD3 and CXCR5. A CXCR5-independent gating strategy based on CD3 and CD20 was applied to determine the percentage of tumor cells and T cells for the co-cultures of (**a**) FL #85, #1100, #1129, (**b**) CLL #47, #12, #31, #33 and (**c**) MCL #114, #578, #624, #988, and the corresponding JeKo-1 controls. Results using the two gating strategies were pooled to generate Fig. 3f-h. (**d, e**) Representative examples of the gating strategy to determine the percentage of $PD1^{+}CXCR5^{+}$ Tfh cells in the $CD4^{+}CD25^{-}$ population for the co-cultures of (**d**) CLL #12, #31, #33, #47, #75 and of (**e**) FL #252, #457, #563, #1054.

Supplementary Figure 9

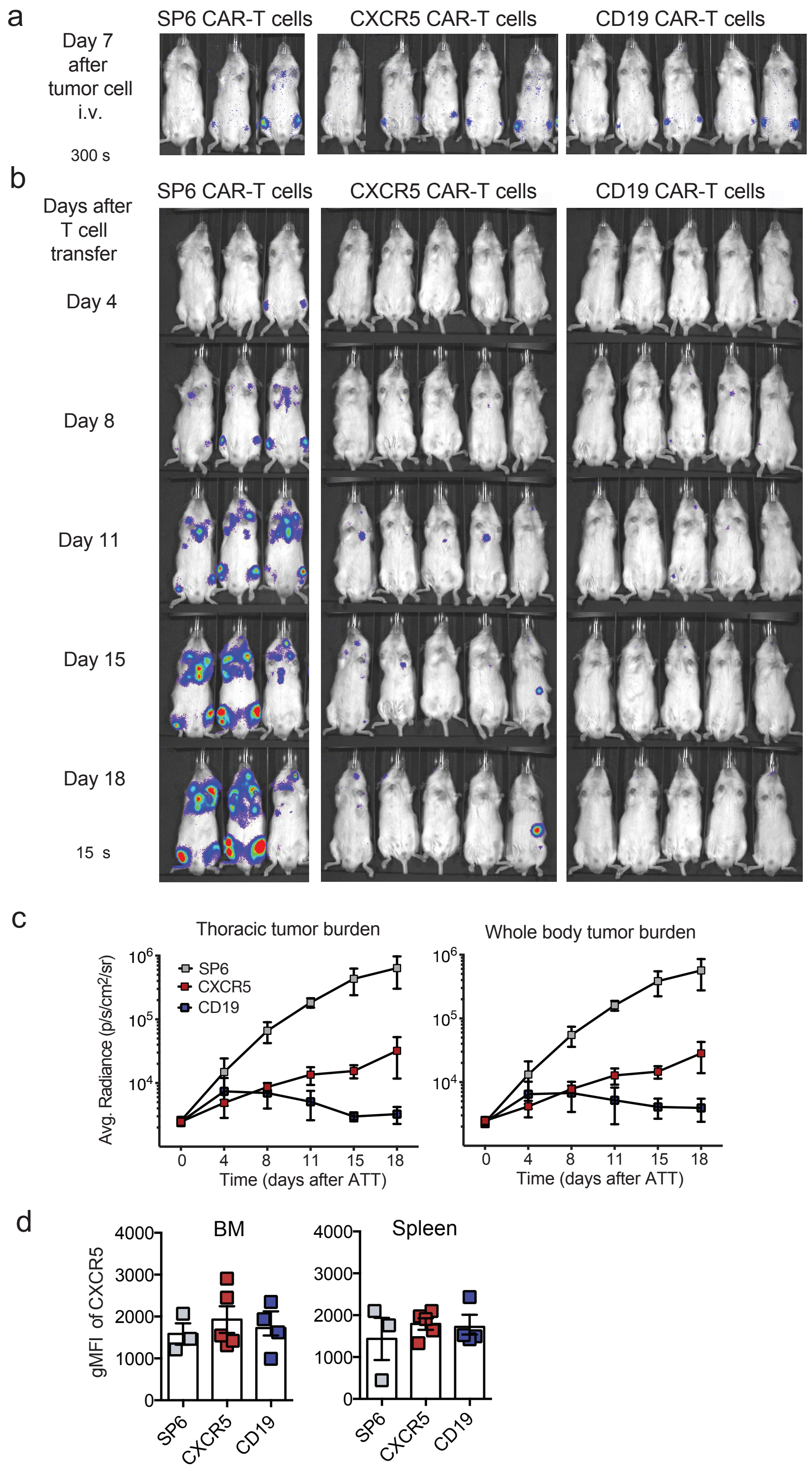

Supplementary Figure 9. Comparable *in vivo* anti-lymphoma efficacy of CXCR5 versus CD19 CAR-T cells.

(**a**) As in Fig. 5, growth of the MCL cell line JeKo-1 ($GFP^{+}luc^{+}$) is shown by bioluminescence imaging at day 7 after tumor inoculation (exposure: 300 s) (**b**) Tumor-bearing mice received $3x10^{6}$ CAR-transduced T cells (total T cells: 4-6 x 10^{6}) and were serially imaged (exposure: 15 s) from day 4 to 18 post T cell infusion (SP6 CAR: n=3; CXCR5 CAR: n=5; CD19 CAR: n=5). (**c**) Means of the signal intensities obtained from the entire body or of thoracic tumor burden are plotted for each group and time point. (**d**) CXCR5 expression on JeKo-1 tumor cells derived from BM or spleen at day 19 or 20 after CAR-T cell transfer. Geometric mean fluorescence intensities (gMFI) of CXCR5 on JeKo-1 tumor cells are shown. Bars represent means ±SEM of n=3 mice (SP6 CAR), n=5 mice (CXCR5 CAR), and n=4 mice (CD19 CAR). Source data are provided as a Source Data file.

Supplementary Figure 10

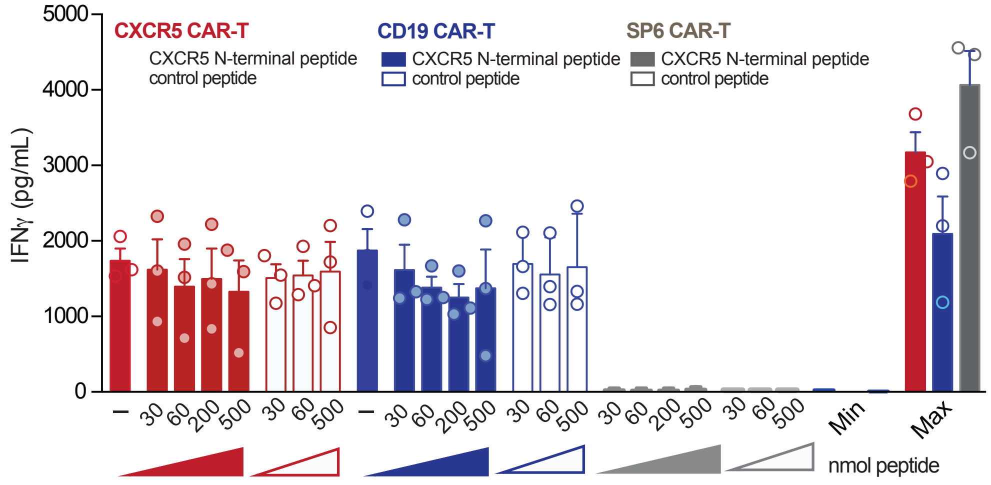

Supplementary Figure 10. CXCR5 CAR-T cells do not show reduced *in vitro* activity upon incubation with a peptide containing the CAR epitope.

CXCR5, CD19 and SP6 CAR transduced cells were co-cultured with JeKo-1 lymphoma cells at a 1:1 ratio for 24 hours. At the start of the culture, a CXCR5 N-terminal peptide containing the CAR epitope or a control peptide of the CXCR5 loop region were added at various concentrations. IFN in the supernatant was determined by ELISA. Max, CAR-T cells with PMA/Ionomycin; Min, CAR-T cells only. Bars represent mean±SEM of 3 independent donors. Source data are provided as a Source Data file.

Supplementary Figure 11

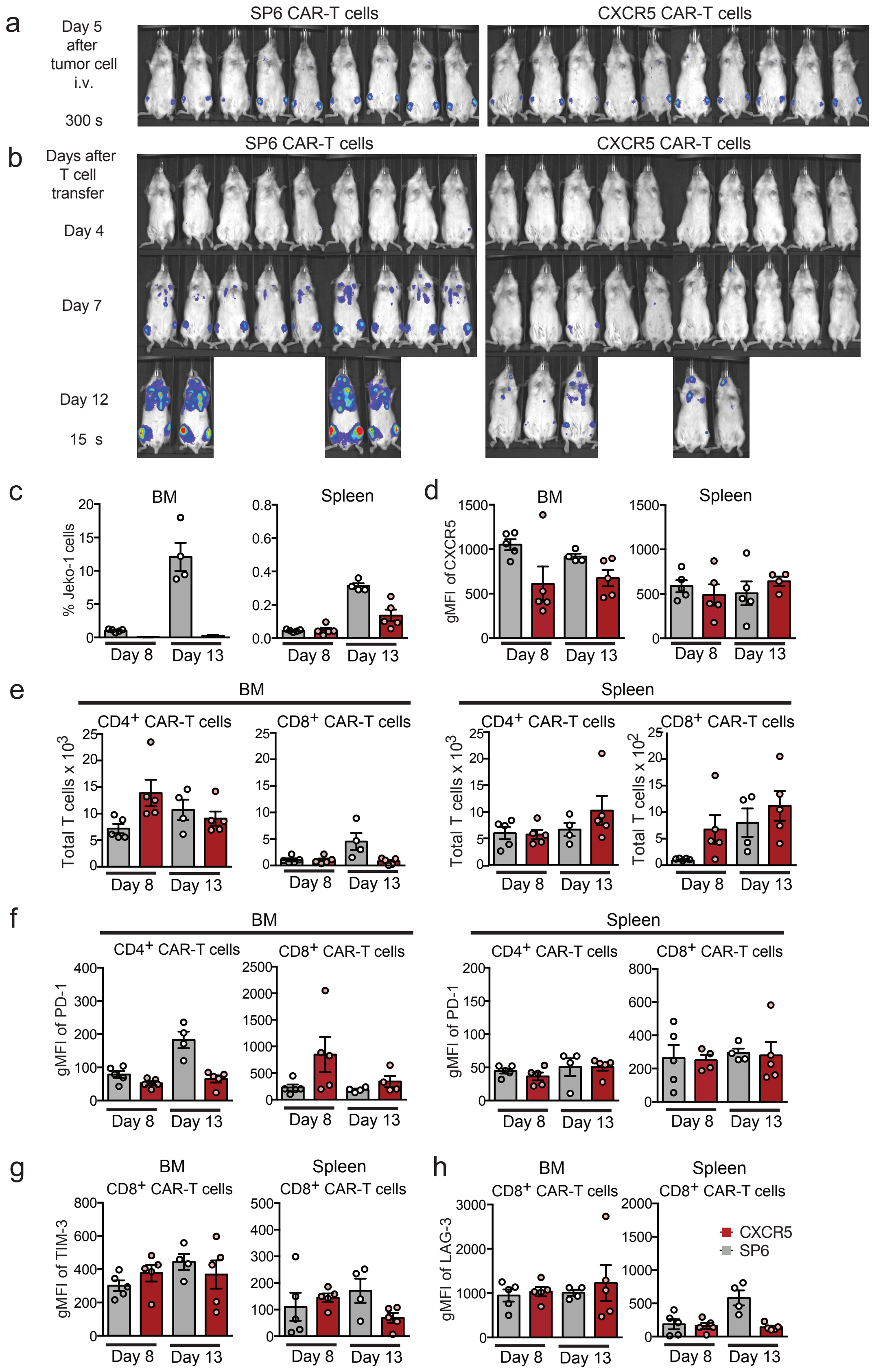

Supplementary Figure 11. CXCR5 CAR T cell persistence and exhaustion marker status *in vivo*. (**a**) NSG mice transplanted with the MCL cell line JeKo-1 were treated with (**b**) $2x10^6$ CAR-transduced T cells (day 5, total T cells: $5\text{-}6x10^6$). Serial IVIS-exposures after CAR-T cell transfer (SP6, n=9; and CXCR5, n=10) were done between day 0 and day 12. (**c-h**) Graphs show the SP6 group in grey and the CXCR5 group in red for two timepoints. At day 8, n=5 animals of both groups, SP6 and CXCR5, were sacrificed. At day 13, n=4 animals of the SP6 group and n=5 animals of the CXCR5 group were sacrificed. At both timepoints, (**c**) JeKo-1 tumor load ($CD19^+$ GFP^+) and (**d**) CXCR5 expression ($CD19^+CXCR5^+$) on JeKo-1 tumor cells in BM and spleen were determined by flow cytometry. (**e**) CAR-T cell numbers ($CD3^+$ $CD8^-$, $CD3^+$ $CD8^+$) in BM and spleen were assessed by flow cytometry in both groups. (**f**) Anti PD-1 costaining for the detection of $CD4^+$ and $CD8^+$ T cell exhaustion; (**g**) LAG-3 and (**h**) TIM-3 costaining for the detection of $CD8^+$ T cell exhaustion. No LAG-3 and TIM-3 staining was detectable on $CD4^+$ T cells. Values depicted in **d**, **f**, **g** and **h** are geometric mean fluorescence intensities (gMFI). The gMFIs of the isotype staining were subtracted from the gMFIs of the specific staining. Bars show the mean ±SEM. Source data are provided as a Source Data file.

Supplementary Figure 12

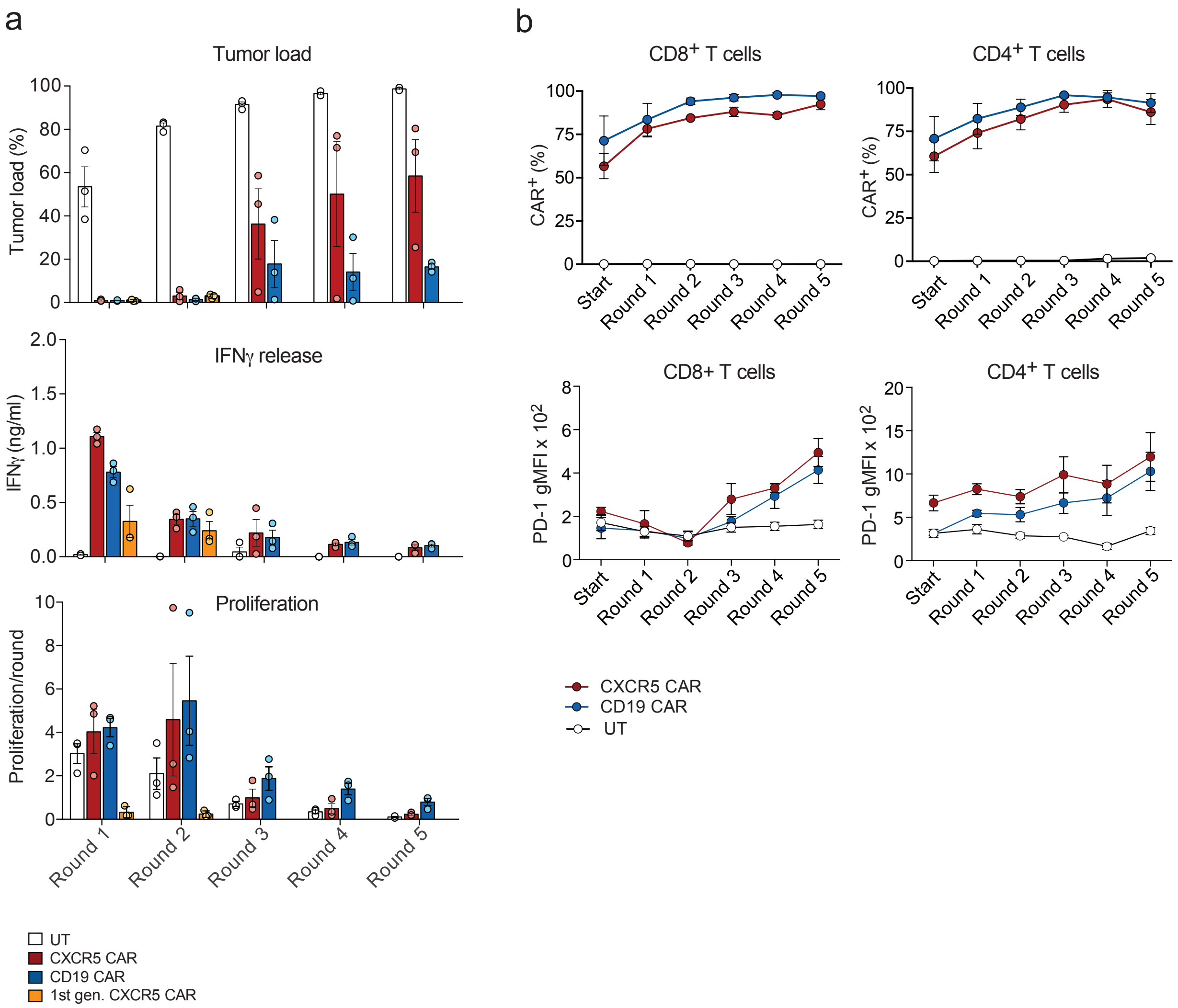

Supplementary Figure 12. In vitro assay of recursive antigen exposure.
(**a**) CXCR5 and CD19 CAR- T cells at day 10 after start of cultivation were co-cultured with JeKo-1 cells in an E:T ratio of 1:2. After 72h, repetitive transfers of CAR-T cells into wells with fresh target cells were performed for a total of five rounds re-establishing the initial E:T ratios. Untransduced (UT) T cells served as controls. A 1st gen. CXCR5 CAR was included for comparison. Three parameters were determined at the end of each round: (i) tumor load (7-AAD$^-$/CXCR5$^+$CD3$^-$) as determined by flow cytometry, (ii) IFNγ release as determined by ELISA, (iii) proliferation of T cells per round (n cells start/ n cells end). (**b**) shows the percentage of CAR$^+$-T subsets and shows the expression of PD-1 on CAR$^+$-T cell subsets. Isotype geometric mean fluorescence intensities (gMFI) were subtracted from the gMFIs of the specific staining. Data in (**a and b**) are pooled from n=2 independent experiments and n=3 individual donors; Bars represent means, errors are SEM. Source data are provided as a Source Data file.

Supplementary Figure 13

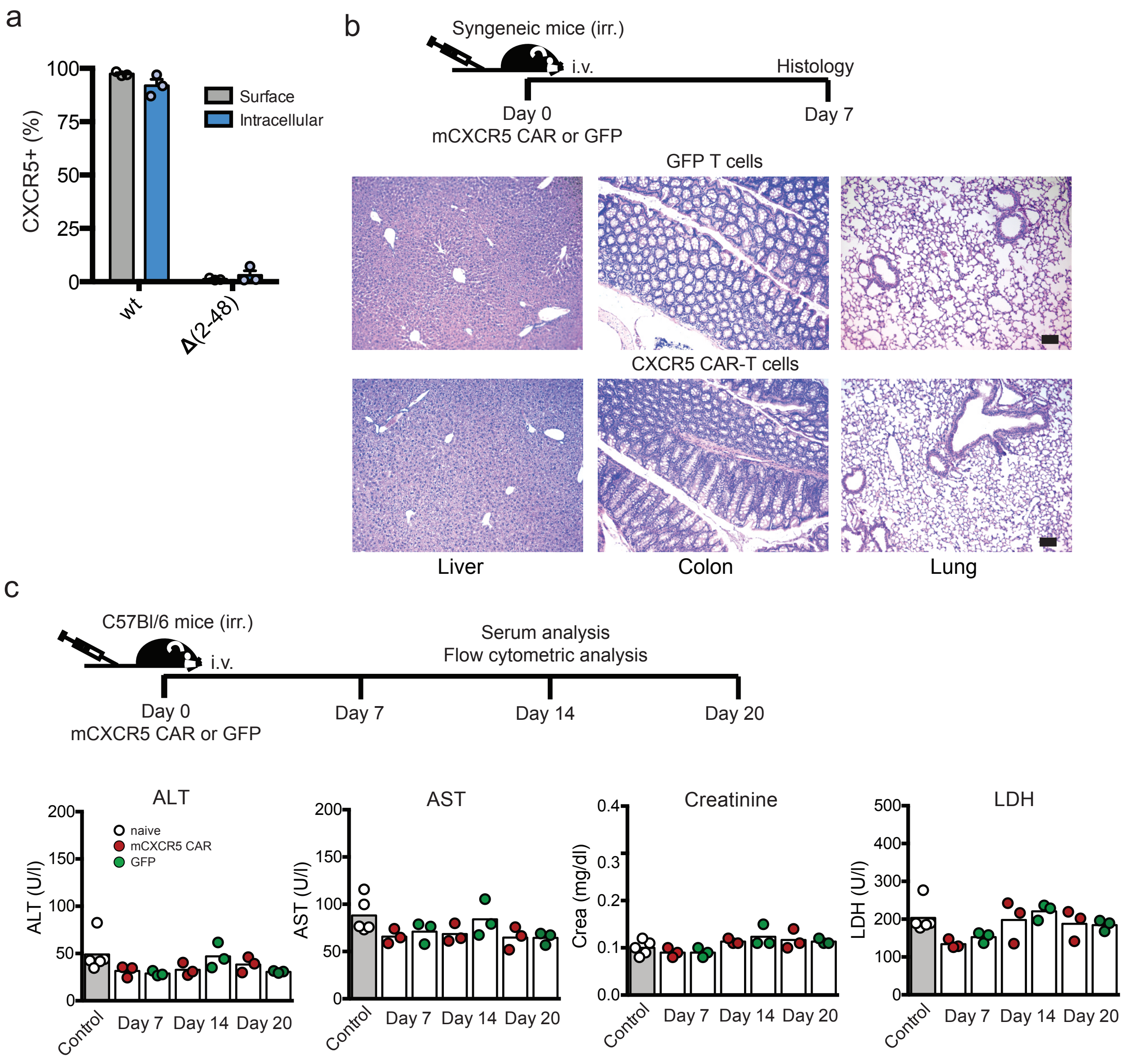

Supplementary Figure 13. Anti-murine CXCR5 CAR-T cells show no off-target reactivity in C57BL/6 mice.

(**a**) The epitope of the rat anti-murine CXCR5 2G8 mAb was mapped using Jurkat cell lines expressing murine CXCR5 deletion variants. Deletions were located in the extracellular N-terminus (2-48) as indicated. CXCR5-negative Jurkat cells were transduced with the murine CXCR5 variants and GFP as a transduction marker, stained with the 2G8 mAb for surface and intracellular protein, and analyzed by flow cytometry. The percentage of GFP-positive, transduced cells that were stained by the mAb are shown. Jurkat cells expressing the unmodified wildtype CXCR5 (wt) served as positive control. Bars represent means ± SEM, and graph shows data of n=3 independent experiments. **(b)** C57BL/6 mice were sublethally irradiated (4.5 Gray; irr.) and 6 hours later $1x10^6$ mCXCR5 CAR- or GFP-transduced T cells were injected i.v. Seven days later the mice were sacrificed and serum and various tissues were retrieved. No inflammatory infiltrates were detected in liver, lung, and colon seven days after mCXCR5-CAR T cell transfer. Depicted are hematoxylin and eosin (H&E) stainings of representative histological sections of liver, lung, and colon of n=3 mice per group. Scale bars, 100 µM. (**c**) C57BL/6 mice were sublethally (4-4.5 Gy) irradiated, followed by i.v. administration of mCXCR5 CAR- or GFP-transduced T cells 4-6h later in two consecutive experiments: i) $1x10^6$ transduced T cells were transferred (n=3 mice per group), treated mice and n=2 naive mice were sacrificed at day 7; ii) $2x10^6$ transduced T cells were transferred (n=6 mice per group). Three mice were sacrificed at day 14 and 20, respectively, naive animals served as controls (n=4). Quantification of serum markers indicative of liver (ALT, AST) and kidney damage (Crea). LDH is a systemic marker for cell damage. Bars represent means. Data of n=2 experiments with n=3 animals per treatment group and time point are shown. Source data are provided as a Source Data file.

Supplementary Figure 14

a

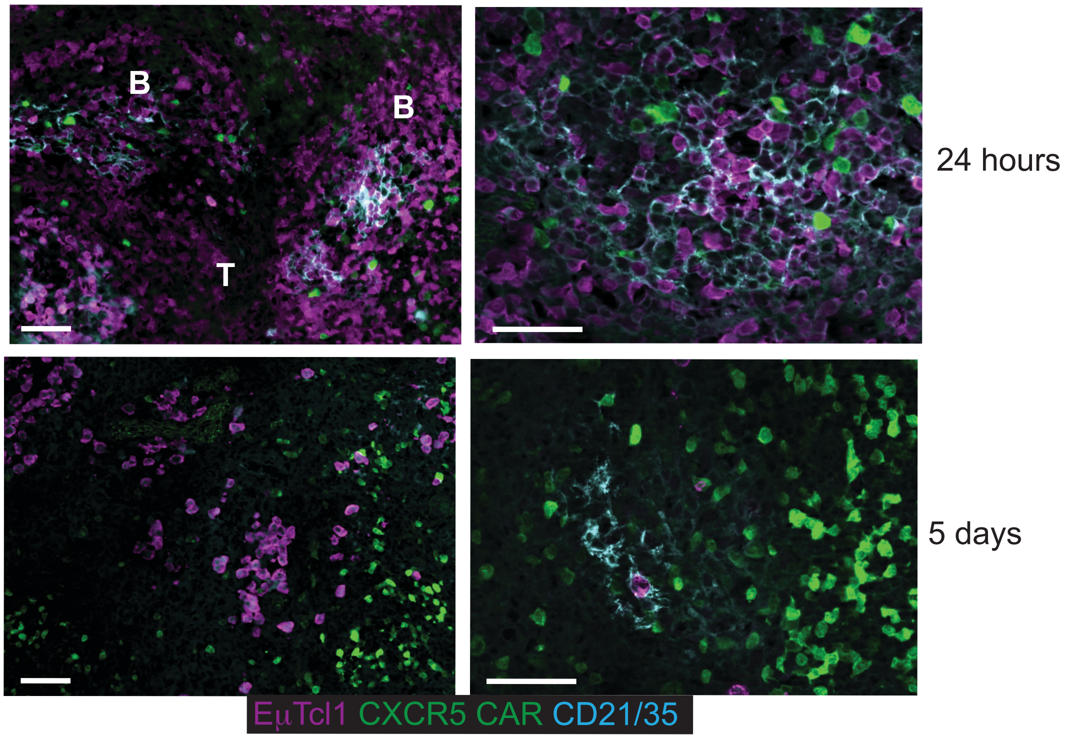

b

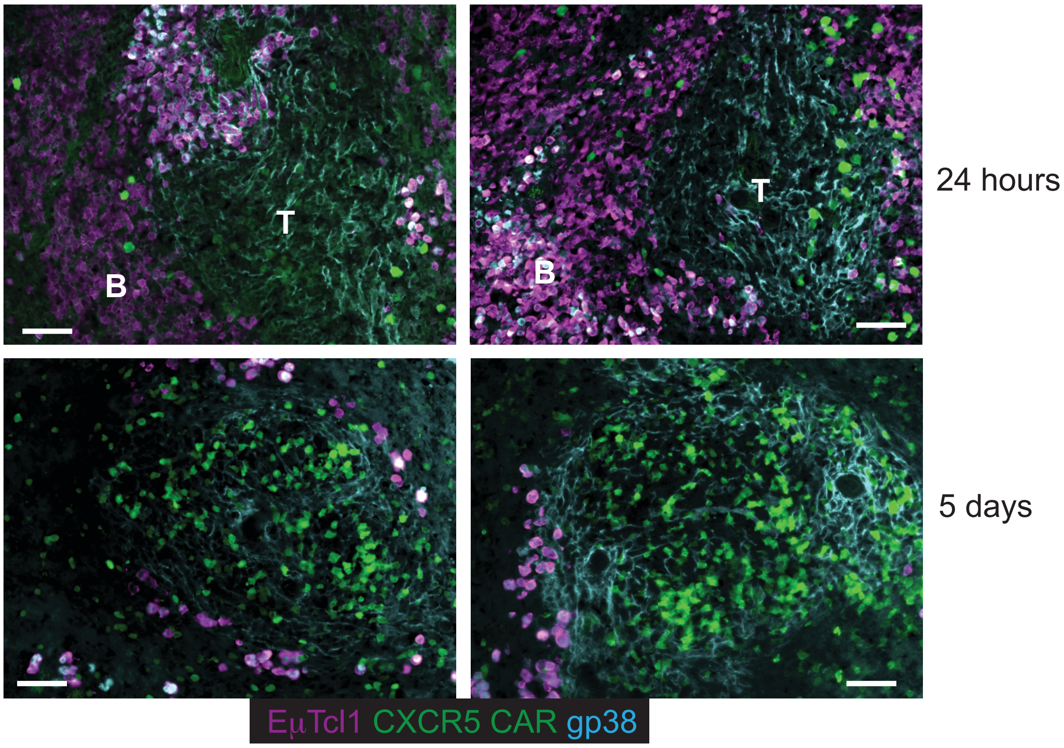

Supplementary Figure 14. Anti-murine CXCR5 CAR-T cells gain access to tumor cells in the B cell follicles and exert potent anti-lymphoma activity.

(**a, b**) Additional histology related to Fig. 9a-e. Congenic mice were injected with $2x10^6$ primary Eμ-Tcl1 lymphoma cells, 8 days later the mice were irradiated (4 Gy) followed 24h later by i.v. administration of $2x10^7$ CAR-T cells expressing mTurquoise2. In two consecutive experiments mice were sacrificed either 24h or 5d thereafter and splenic CAR-T cells and Eμ-Tcl1 lymphoma cells were analyzed by immunohistology (Scale bars, 50 μm). 24-hour group n=3, 5-day group n=4, control n=1 (no CAR-T cells).

Supplementary Table 1: List of DNA primers

Primer	Sequence (5'->3')	Notes
WPRE FWD	gag gag ttg tgg ccc gtt gt	Copy number determination of retroviral insertion
WPRE REV	tga cag gtg gtg gca atg cc	
WPRE probe	FAM-ctg tgt ttg ctg acg caa c-BHQ1	
PTBP2 FWD	tct cca ttc cct atg ttc atg c	
PTBP2 probe	gtt ccc gca gaa tgg tga ggt g	
PTBP2 REV	JOE-atg ttc ctc gga cca act tg-BHQ1	
MP71-5UTR FWD	cag cat cgt tct gtg ttg tct	Sequencing and general cloning
MP71-3UTR REV	tga ttg ccc cac cat ttt gt	Sequencing and general cloning
huCXCR5-NotI FWD	tta cag gcg gcc gcc acc atg aac tac ccg cta acg ctg	Cloning of human CXCR5 (wt) into the SB transposon vector via NotI/EcoRI and general huCXCR5 cloning
huCXCR5-EcoRI REV	tgc tcg aat tcc tag aac gtg gtg aga gag gtg gc	
huCXCR5-P2A-GFP REV	gct gaa gtt ggt ggc gcc gct gcc gaa cgt ggt gag aga ggt ggc	Generation of huCXCR5 (wt)-P2A-GFP and deletion variants by overlap extension PCR and cloning into MP71 via NotI/EcoRI
huCXCR5-P2A-GFP FWD	gcc acc tct ctc acc acg ttc ggc agc ggc gcc acc aac ttc agc	
GFP-EcoRI REV	tgc tcg aat tct tac ttg tac agc tcg tcc atg c	
huCXCR5-Δ(2-48) FWD	tta cag gcg gcc gcc acc atg gcc tcc ttc aag gcc gtg	Generation of huCXCR5-Δ(2-48)-P2A-GFP
huCXCR5-Δ(2-8) FWD	ttg gcg gcc gcc acc atg gac ctc gag aac ctg gag gac c	Generation of huCXCR5-Δ(2-8)-P2A-GFP
huCXCR5-Δ(2-18) FWD	ttg gcg gcc gcc acc atg ttc tgg gaa ctg gac aga ttg g	Generation of huCXCR5-Δ(2-18)-P2A-
huCXCR5-Δ(22-45) FWD	gga gga cct gtt ctg gga act gat ggc ctc ctt caa ggc cgt g	Generation of huCXCR5-Δ(22-45)-P2A-GFP
huCXCR5-Δ(22-45) REV	cac ggc ctt gaa gga ggc cat cag ttc cca gaa cag gtc ctc c	
huCXCR5-Δ(30-45) FWD	cag att gga caa cta taa cga cat ggc ctc ctt caa ggc cgt g	Generation of huCXCR5-Δ(30-45)-P2A-GFP
huCXCR5-Δ(30-45) REV	cac ggc ctt gaa gga ggc cat gtc gtt ata gtt gtc caa tct g	
huCXCR5-Δ(38-45) FWD	ctc cct ggt gga aaa tca tct cat ggc ctc ctt caa ggc cgt g	Generation of huCXCR5-Δ(38-45)-P2A-GFP
huCXCR5-Δ(38-45) REV	cac ggc ctt gaa gga ggc cat gag atg att ttc cac cag gga g	
CD28tm-CD3z REV	ctg ctg aac ttc act ctc agc acc cag aaa ata ata aag gcc act g	Generation of 1st gen. huCXCR5 CAR
CD28tm-CD3z FWD	cct tta tta ttt tct ggg tgc tga gag tga agt tca gca gga gc	
muCXCR5-NotI FWD	tta cag gcg gcc gcc acc atg aac tac cca cta acc ctg g	Generation of muCXCR5 (wt)-P2A-GFP and muCXCR5 Δ(2-48)-P2A-GFP by overlap extension PCR and cloning into MP71 via NotI/MfeI
muCXCR5-P2A REV	gct gaa gtt ggt ggc gcc gct gcc gaa ggt ggt gag gga agt agc	
muCXCR5-P2A FWD	gct act tcc ctc acc acc ttc ggc agc ggc gcc acc aac ttc agc	
P2A-GFP-MfeI REV	tgc tca att gtt act tgt aca gct cgt cca tgcc	
muCXCR5-Δ(2-48) FWD	tta cag gcg gcc gcc acc atg acg tcc ttt aag gcg gta ttc	
