# Supplementary material for: CXCR5 CAR-T cells simultaneously target B cell non-Hodgkin’s lymphoma and tumor-supportive follicular T helper cells
Source: Nat Commun. 2021 Jan 11;12:240. doi: 10.1038/s41467-020-20488-3 (PMC7801647; doi:10.1038/s41467-020-20488-3)
Supplement: Supplementary file 3 — Reporting Summary [file 41467_2020_20488_MOESM3_ESM.pdf]

nature research

Corresponding author(s): Uta E. Höpken

Last updated by author(s): Nov 27, 2020

Reporting Summary

Nature Research wishes to improve the reproducibility of the work that we publish. This form provides structure for consistency and transparency in reporting. For further information on Nature Research policies, see our Editorial Policies and the Editorial Policy Checklist.

Statistics

For all statistical analyses, confirm that the following items are present in the figure legend, table legend, main text, or Methods section.

n/a	Confirmed	
☐	☒	The exact sample size (n) for each experimental group/condition, given as a discrete number and unit of measurement
☐	☒	A statement on whether measurements were taken from distinct samples or whether the same sample was measured repeatedly
☐	☒	The statistical test(s) used AND whether they are one- or two-sided<br>*Only common tests should be described solely by name; describe more complex techniques in the Methods section.*
☒	☐	A description of all covariates tested
☒	☐	A description of any assumptions or corrections, such as tests of normality and adjustment for multiple comparisons
☐	☒	A full description of the statistical parameters including central tendency (e.g. means) or other basic estimates (e.g. regression coefficient) AND variation (e.g. standard deviation) or associated estimates of uncertainty (e.g. confidence intervals)
☐	☒	For null hypothesis testing, the test statistic (e.g. F, t, r) with confidence intervals, effect sizes, degrees of freedom and P value noted<br>*Give P values as exact values whenever suitable.*
☒	☐	For Bayesian analysis, information on the choice of priors and Markov chain Monte Carlo settings
☒	☐	For hierarchical and complex designs, identification of the appropriate level for tests and full reporting of outcomes
☒	☐	Estimates of effect sizes (e.g. Cohen's d, Pearson's r), indicating how they were calculated

Our web collection on statistics for biologists contains articles on many of the points above.

Software and code

Policy information about availability of computer code

Data collection	FACS Diva version 6.1.3 (BD), Living image 4.5 (Perkin Elmer), Axio Vision 4.8.2 software (Carl Zeiss), KC4 V3.0 (Bio-TEK), SpectroFlo version 2.2.0 (Cytek)
Data analysis	GraphPad Prism 7, FlowJo 10 (BD), Excel 14.7.3 (Microsoft), Genevestigator V7.4.1 (Nebion), FCS Express 7.00.0037 Research (De Novo Software)

For manuscripts utilizing custom algorithms or software that are central to the research but not yet described in published literature, software must be made available to editors and reviewers. We strongly encourage code deposition in a community repository (e.g. GitHub). See the Nature Research guidelines for submitting code & software for further information.

Data

Policy information about availability of data

All manuscripts must include a data availability statement. This statement should provide the following information, where applicable:

- Accession codes, unique identifiers, or web links for publicly available datasets
- A list of figures that have associated raw data
- A description of any restrictions on data availability

The data that support the findings of this study are available from the corresponding author upon reasonable request. The source data for the Figs. 1b, 1d, 1e, 2c-g, 3a-i, 4c-d, 5c, 6c-d, 7b-d, 8c-e, 8g-i, 9c-e, 9g-h and Supplementary Figs. S5a-b, S6, S7b-c,S9c-d, S10, S11c-h, S12a-b, S13c are provided as a Source Data file. Reference sequences of human CD28 (NM_006139.4), CD3z (NM_198053.3), CXCR5 (NM_001716.5) and mouse Cxcr5 (NM_007551.3) were downloaded from the NCBI RefSeq database (https://www.ncbi.nlm.nih.gov/refseq/). The data underlying Supplementary Fig. S3 was accessed from the HS_AFFY_U133PLUS_2-0 database using the free basic edition of the Genevestigator (Nebion) software (https://genevestigator.com) on November 4, 2019.

Field-specific reporting

Please select the one below that is the best fit for your research. If you are not sure, read the appropriate sections before making your selection.

☒ Life sciences ☐ Behavioural & social sciences ☐ Ecological, evolutionary & environmental sciences

For a reference copy of the document with all sections, see nature.com/documents/nr-reporting-summary-flat.pdf

Life sciences study design

All studies must disclose on these points even when the disclosure is negative.

Sample size	The chosen sample sizes were based on pilot experiments and our prior experiences from studying an anti-BCMA CAR in comparable experimental systems (Bluhm et al., Mol Ther. 2018 Aug 1; 26(8): 1906–1920). According to our experiences with in vivo animal models of CAR-T cell therapy, a treatment group of four to six animals would likely enable detection of statistically significant differences compared to an untreated or mock-treated control group. For in vitro experiments with human CAR-T cells, a minimum of three donors were used, but most data represents more donors and experiments.
Data exclusions	No data was excluded.
Replication	All in vitro experiments were reliably reproduced. Data of individual donors and from independent experiments were pooled. The numbers of donors and experiments were indicated in the figure legends. A minimum of three donors was pooled from at least two independent experiments, but most data represents more donors and experiments. All co-culture experiments with human CAR-T cells were performed with technical duplicates. In vivo animal models were conducted at least twice, unless specified exactly as in figure legends. Treatment groups were four to six animals. Control groups were always included. CAR T cell-treated animals showed reproducible suppression of tumor growth and/or B cell depletion across the experiments.
Randomization	Blood donors for the CAR-T cells were selected randomly. Primary tumor material was from random patients with a specific cancer type. For in vivo models without tumor, mice were assigned randomly to experimental and control groups. For in vivo tumor models, mice were analyzed and distributed before CAR-T cell transfer so that the overall tumor burden was consistent across the experimental groups.
Blinding	In general, experiments were not blinded. Blinding was not strictly required because the reported outcomes are based on non-subjective measurements. Experiments were designed so that multiple samples were prepared and measured side-by-side in a highly uniform manner. Appropriate controls were always included.

Reporting for specific materials, systems and methods

We require information from authors about some types of materials, experimental systems and methods used in many studies. Here, indicate whether each material, system or method listed is relevant to your study. If you are not sure if a list item applies to your research, read the appropriate section before selecting a response.

Materials & experimental systems

n/a	Involved in the study	
☐	☒	Antibodies
☐	☒	Eukaryotic cell lines
☒	☐	Palaeontology and archaeology
☐	☒	Animals and other organisms
☐	☒	Human research participants
☒	☐	Clinical data
☒	☐	Dual use research of concern

Methods

n/a	Involved in the study	
☒	☐	ChIP-seq
☐	☒	Flow cytometry
☒	☐	MRI-based neuroimaging

Antibodies

Antibodies used	Antibodies were purchased from Biolegend/Biozol (München) unless stated otherwise. Surface expression of human CARs was determined using a polyclonal FITC or PE-labeled goat anti-human IgG (#2040-09, Southern Biotech/Biozol, FITC 1:400, PE 1:600) or biotin anti-human IgG (IS11-12E4.23.20, #130-119-858, Miltenyi Biotec, 1:10). The cells were washed twice before subsequent costaining. Costainings were performed using the following mAbs: PB anti-CD3 (HIT3a, #300330, 1:100); BV421 anti-CD4 (RPA-T4, #300532, 1:200) or APC/Cy7 anti-CD4 (OKT4, #317450, 1:100); AF647 anti-LAG-3 (11C3C65, #369304, 1:50); PE anti-PD-1 (EH12.2H7, #329906, 1:50); BV421 anti-TIM-3 (F38-2E2, #345008, 1:50); PB anti-CD8 (HIT8a, #300928, 1:100), APC anti-CD8 (HIT8a, #300912, 1:100) or PE/Cy7 anti-CD8 (HIT8a, 300914, 1:100); FITC anti-CD45RO (UCHL1,#304242, 1:200); PerCP/Cy5.5 anti-CD45RA (HI100, #304122, 1:200); PE/Cy7 anti-CD197 (G043H7, #353226, 1:200); AF647 anti-CD62L (DREG-56, #304818, 1:200); AF647 anti-CXCR5 (RF8B2, #558113, BD Bioscience, 1:100). Tumor cell lines were stained with PE anti-CXCR5 (51505, #FAB190P-100, R&D Systems/Bio-Techne, 1:10), PB anti-CD19 (HIB19, #302224, 1:200) or BV510 anti-CD19 (SJ25C1, #363020, 1:100). Patient biopsies were analyzed using two mAb cocktails: MZL and FL samples were stained with PE anti-CXCR5 (51505, #FAB190P-100, R&D Systems/Bio-Techne, 1:10), BV510 anti-CD19 (SJ25C1, #363020, 1:100) and PB anti-CD3 (HIT3a, #300330, 1:100). CLL and MCL samples were stained with

PE anti-CXCR5 (51505, #FAB190P-100, R&D Systems/Bio-Techne, 1:10), PB anti-CD5 (UCHT2, #300624, 1:165), FITC anti-CD3 (HIT3a, #300306, 1:100), and APC anti-CD20 (2H7, #302310, 1:100). PBMCs were analyzed with two mAb panels: 1) lymphocytes: FITC anti-CD4 (OKT4, 1:100), PE anti-CXCR5 (51505, #FAB190P-100, R&D Systems/Bio-Techne, 1:10), PE/Cy7 anti-CD56 (5.1H11, #362510, 1:100), APC anti-CD25 (BC96, #302610, 1:100), APC/Fire750 anti-CD8 (SK1, #344746, 1:100), BV421 anti-PD-1 (EH12.2H7, #329920, 1:50) and BV510 anti-CD19 (SJ25C1, #363020, 1:100). 2) Monocytes and DCs: FITC anti-CD1c (L161, #331518, 1:100), PE anti-CXCR5 (51505, #FAB190P-100, R&D Systems/Bio-Techne, 1:10), PE/Cy7 anti-CD11c (Bu15, #337216, 1:100), APC anti-CD14 (HCD14, #325608, 1:100), APC/Fire750 anti-CD303 (201A, #354236, 1:100), BV421 anti-HLA-DR (L243, #307636, 1:100), BV510 anti-CD19 (SJ25C1, #363020, 1:100) and BV510 anti-CD3 (SK7, #344828, 1:100).

The mouse CAR was detected using polyclonal PE-labeled goat anti-mouse IgG-Ab (#1030-09, Southern Biotech/Biozol, 1:200). Splenocytes of mice were additionally stained with PE-Cy7 anti-PD1 (RMP1-30, #109110, 1:100), APC anti-CD3 (145-2C11, #100312, 1:200), APC/Fire anti-CD4 (RM4-5, #100568, 1:200), BV421 anti-CXCR5 (L138D7, #145512, 1:200) and V500 anti-B220 (RA3-6B2, #561226, BD Bioscience, 1:200). In the therapeutic tumor mouse model, the CAR-T cells were detected using monoclonal PE/Cy7 anti-mouse IgG1 (RMG1-1, #406613, 1:200). Costainings were performed using AF488 anti-CD45.2 (104, #109816, 1:200), APC/Fire anti-CD4 (RM4-5, #100568, 1:200) and PB anti-CD8 (53-6.7, #100725, 1:200). Eμ-Tcl1 tumor cells in the spleen were analyzed using FITC anti-CD45.1 (A20, #109816, 1:200), PE anti-CD5 (53-7.3, #100608, 1:200), APC anti-CD3e (145-2C11, #100312, 1:200), BV421 anti-CXCR5 (L138D7, #145512, 1:200) and BV510 anti-CD19 (6D5, #115546, 1:200). Blood samples from the mouse immunization experiment were stained with PE/Cy7 anti-mouse IgG1 (RMG1-1, #406613, 1:200), APC/Cy7 anti-CD45.2 (104, #109824, 1:200), AF700 anti-CD8 (53-6.7, #557959, BD Bioscience, 1:200), SparkBlue550 anti-CD4 (GK1.5, #100474, 1:400), BV421 anti-CXCR5 (L138D7, #145512, 1:100), BV510 anti-CD19 (6D5, #115546, 1:200) and BUV395 anti-CD3 (145-2C11, #565992, BD Bioscience, 1:200). The antigen-specific T cells were detected using a PE H-2kb/VVYDFLKL (SV40 Tag IV) dextramer (Immudex) and FITC anti-CD45.1 (A20, #109816, 1:200), APC/Cy7 anti-B220 (RA3-6B2, #103224, 1:200) and PB anti-CD8 (KT15, #MCA609PB, Bio-Rad, 1:200).

Validation

All antibodies used in this study were commercially available and validated by the manufacturer for flow cytometry. In addition, we used antigen-positive and -negative cells as controls, and/or isotype controls to ensure that only antigen-specific staining is observed. In the following, we provide links to the product webpages of the manufacture where more details and references are provided. Polyclonal goat anti-human IgG serum (#2040-09 Southern Biotech) binds to human IgG in an FLISA and was validated for flow cytometry (https://www.southernbiotech.com/?catno=2040-09&type=Polyclonal#&panel2-1). It detects CARs with human IgG1 spacer on transduced human T cells as demonstrated in our present study (Fig. 2) and in our previous BCMA CAR study (PMID: 30078440). The mouse anti-human IgG antibody (IS11-12E4.23.20) from Miltenyi Biotec detects IgG on B cells of human PBMCs and was validated for flow cytometry: https://www.miltenyibiotec.com/DE-en/products/igg1-antibody-anti-human-is11-12e4-23-20.html#biotin:100-tests-in-200-ul. The mouse anti-human CD1c antibody (L161) from Biolegend was validated by the manufacturer for flow cytometry using MOLT4 cells: https://www.biolegend.com/en-us/products/fitc-anti-human-cd1c-antibody-6602. The mouse anti-human CD3 antibody (HIT3a) from Biolegend was validated by the manufacturer for flow cytometry using human PBMCs: https://www.biolegend.com/en-us/products/fitc-anti-human-cd3-antibody-751. The mouse anti-human CD3 antibody (SK7) from Biolegend was validated by the manufacturer for flow cytometry using human PBMCs: https://www.biolegend.com/en-us/products/fitc-anti-human-cd3-antibody-751. The mouse anti-human CD4 antibody (RPA-T4) from Biolegend was validated for flow cytometry by the manufacturer using human PBMCs: https://www.biolegend.com/en-us/products/brilliant-violet-421-anti-human-cd4-antibody-7151. The mouse anti-human CD4 antibody (OKT4) from Biolegend was validated by the manufacturer for flow cytometry using human PBMCs: https://www.biolegend.com/en-us/products/apc-cyanine7-anti-human-cd4-antibody-3658. The mouse anti-human CD5 antibody (UCHT2) from Biolegend was validated by the manufacturer for flow cytometry using human PBMCs: https://www.biolegend.com/en-us/products/apc-cyanine7-anti-human-cd4-antibody-3658. The mouse anti-human CD8 antibody (HIT8a) from Biolegend was validated by the manufacturer for flow cytometry using human PBMCs: https://www.biolegend.com/en-us/products/apc-anti-human-cd8a-antibody-759. The mouse anti-human CD8 antibody (SK1) from Biolegend was validated by the manufacturer for flow cytometry using human PBMCs: https://www.biolegend.com/en-us/products/apc-fire-750-anti-human-cd8-antibody-13035. The mouse anti-human CD11c antibody (Bu15) from Biolegend was validated by the manufacturer for flow cytometry using human peripheral blood monocytes: https://www.biolegend.com/en-us/products/pe-cyanine7-anti-human-cd11c-antibody-6129. The mouse anti-human CD14 antibody (HCD14) from Biolegend was validated by the manufacturer for flow cytometry using human peripheral blood monocytes: https://www.biolegend.com/en-us/products/apc-anti-human-cd14-antibody-3953. The mouse anti-human CD19 antibody (HIB19) from Biolegend was validated by the manufacturer for flow cytometry using human PBMCs: https://www.biolegend.com/en-us/products/pacific-blue-anti-human-cd19-antibody-3334. The mouse anti-human CD19 antibody (SJ25C1) from Biolegend was validated by the manufacturer for flow cytometry using human PBMCs: https://www.biolegend.com/en-us/products/brilliant-violet-510-anti-human-cd19-antibody-10334. The mouse anti-human CD20 antibody (2H7) from Biolegend was validated by the manufacturer for flow cytometry using human PBMCs: https://www.biolegend.com/en-us/products/apc-anti-human-cd20-antibody-14562. The mouse anti-human CD25 antibody (BC967) from Biolegend was validated by the manufacturer for flow cytometry using PHA-stimulated (3 day) human PBMCs: https://www.biolegend.com/en-us/products/apc-anti-human-cd25-antibody-614. The mouse anti-human CD45RO antibody (UCHL1) from Biolegend was validated by the manufacturer for flow cytometry using human PBMCs: https://www.biolegend.com/en-us/products/fitc-anti-human-cd45ro-antibody-857. The mouse anti-human CD45RA antibody (HI100) from Biolegend was validated by the manufacturer for flow cytometry using human PBMCs: https://www.biolegend.com/en-us/products/percp-cyanine5-5-anti-human-cd45ra-antibody-4241. The mouse anti-human CD56 antibody (5.1H11) from Biolegend was validated by the manufacturer for flow cytometry using human PBMCs: https://www.biolegend.com/en-us/products/pe-cyanine7-anti-human-cd56-ncam-antibody-9959. The mouse anti-human CD62L antibody (DREG-56) from Biolegend was validated by the manufacturer for flow cytometry using human PBMCs: https://www.biolegend.com/en-us/products/alexa-fluor-647-anti-human-cd62l-antibody-3346. The mouse anti-human CD197 antibody (G043H7) from Biolegend was validated by the manufacturer for flow cytometry using human PBMCs: https://www.biolegend.com/en-us/products/pecyanine7-anti-human-cd197-ccr7-antibody-7694. The mouse anti-human CD303 antibody (201A) from Biolegend was validated by the manufacturer for flow cytometry using human PBMCs: https://www.biolegend.com/en-us/products/apcfire750-anti-human-cd303-bdca-2-antibody-16577. The mouse anti-human HLA-DR antibody (L243) from Biolegend was validated by the manufacturer for flow cytometry using human PBMCs: https://www.biolegend.com/en-us/products/brilliant-violet-421-anti-human-hla-dr-antibody-7226. The mouse anti-human LAG-3 (CD223) antibody (11C3C65) from Biolegend was validated by the manufacturer for flow cytometry using CD3/CD28/IL-2 stimulated (three days) human PBMCs: https://www.biolegend.com/en-us/products/alexa-fluor-647-anti-human-cd223-lag-3-antibody-12465. The mouse anti-human PD-1 (CD279) antibody (EH12.2H7) from Biolegend was validated by the manufacturer for flow cytometry using PHA stimulated (three days) human PBMCs: https://www.biolegend.com/en-us/products/pe-anti-human-cd279-pd-1-antibody-4412. The mouse anti-human TIM-3 (CD366) antibody (F38-2E2) from Biolegend was validated by the manufacturer for flow cytometry using Th1-polarized human PBMCs: https://www.biolegend.com/en-us/products/brilliant-violet-421-anti-human-cd366-tim-3-antibody-7401. The rat anti-human CXCR5 (CD185) antibody (RF8B2) from BD Bioscience was validated by the manufacturer for flow

cytometry using human PBMCs: https://www.bdbiosciences.com/us/applications/research/t-cell-immunology/t-follicular-helper-tfh-cells/surface-markers/human/alexa-fluor-647-rat-anti-human-cxcr5-cd185-rf8b2/p/558113. The mouse anti-human CXCR5 (CD185) antibody (51505) from R&D Biosystems was validated by the manufacturer for flow cytometry using human PBMCs: https://www.rndsystems.com/products/human-cxcr5-antibody-51505_mab190.

Polyclonal goat anti-mouse IgG serum (#1030-09 Southern Biotech) binds to mouse IgG in an FLISA and was validated for flow cytometry (https://www.southernbiotech.com/?catno=1030-09&type=Polyclonal#&panel1-1&panel2-1). It detects CARs with mouse IgG1 spacer on transduced mouse T cells as demonstrated in our present study (Fig. 8) and a previous mouse CAR study (PMID: 29241547). The rat anti-mouse IgG1 antibody (RMG1-1) from Biolegend was validated by the manufacturer for flow cytometry using human PBMCs that were first stained with a mouse anti-human CD3 antibody followed by anti-mouse IgG1: https://www.biolegend.com/en-us/products/pe-cyanine7-anti-mouse-igg1-8489. The hamster anti-mouse CD3e antibody (145-2C11) from Biolegend was validated by the manufacturer for flow cytometry using C57BL/6 mouse splenocytes: https://www.biolegend.com/en-us/products/apc-anti-mouse-cd3epsilon-antibody-21. The rat anti-mouse CD4 antibody (RM4-5) from Biolegend was validated by the manufacturer for flow cytometry using C57BL/6 mouse splenocytes: https://www.biolegend.com/en-us/products/apc-fire-750-anti-mouse-cd4-antibody-13560. The rat anti-mouse CD4 antibody (GK1.5) from Biolegend was validated by the manufacturer for flow cytometry using C57BL/6 mouse splenocytes: https://www.biolegend.com/en-us/products/spark-blue-550-anti-mouse-cd4-antibody-18488. The rat anti-mouse CD8a antibody (53-6.7) from Biolegend was validated by the manufacturer for flow cytometry using C57BL/6 mouse splenocytes: https://www.biolegend.com/en-us/products/pacific-blue-anti-mouse-cd8a-antibody-2856. The rat anti-mouse CD8a antibody (KT15) from Biorad was validated by the manufacturer for flow cytometry using mouse blood: https://www.bio-rad-antibodies.com/monoclonal/mouse-cd8-alpha-antibody-kt15-mca609.html?f=purified. The rat anti-mouse CD19 antibody (6D5) from Biolegend was validated by the manufacturer for flow cytometry using C57BL/6 mouse splenocytes: https://www.biolegend.com/en-us/products/brilliant-violet-510-anti-mouse-cd19-antibody-8563. The mouse anti-mouse CD45.1 antibody (A20) from Biolegend was validated by the manufacturer for flow cytometry using SJL mouse splenocytes: https://www.biolegend.com/en-us/products/fitc-anti-mouse-cd45-1-antibody-198. The mouse anti-mouse CD45.2 antibody (104) from Biolegend was validated by the manufacturer for flow cytometry using C57BL/6 mouse splenocytes: https://www.biolegend.com/en-us/products/alexa-fluor-488-anti-mouse-cd45-2-antibody-3106. The rat anti-mouse CXCR5 (CD185) antibody (L138D7) from Biolegend was validated by the manufacturer for flow cytometry using C57BL/6 mouse splenocytes: https://www.biolegend.com/en-us/products/brilliant-violet-421-anti-mouse-cd185-cxcr5-antibody-8553. The rat anti-mouse B220 (CD45R) antibody (RA3-6B2) from Biolegend was validated by the manufacturer for flow cytometry using C57BL/6 mouse splenocytes: https://www.biolegend.com/en-us/products/apc-cyanine7-anti-mouse-human-cd45r-b220-antibody-1938. The rat anti-mouse PD-1 (CD279) antibody (RMP1-30) from Biolegend was validated by the manufacturer for flow cytometry using Con A-stimulated (day-3) Balb/c mouse splenocytes: https://www.biolegend.com/en-us/products/pe-cyanine7-anti-mouse-cd279-pd-1-antibody-3612.

Eukaryotic cell lines

Policy information about cell lines

Cell line source(s)	The human cell lines DOHH-2 (ACC 47), SC-1 (ACC 558), SU-DHL-4 (ACC 495), OCI-Ly7 (AC 688), JEKO-1 (ACC 553), NCI-H929 (ACC 163), RAJI (ACC 319), JURKAT (ACC 282), and HEP-G2 (ACC 180) were obtained from DSMZ (Braunschweig, Germany). The cell line SW-620 (#300466) was obtained from CLS Inc. (Eppelheim, Germany). The cell line HEK293 was purchased from Quantum Biotechnologies Inc. (Quebec, Canada). The cell lines NALM-6 and REH were obtained from Dr. Stephan Mathas (MDC, Berlin, Germany). Primary human umbilical vein endothelial cells (HUVEC, C-12200) and human umbilical arterial endothelial cells (HUAEC, C-12202) were purchased from PromoCell (Heidelberg, Germany). Primary human perineurial cells (HPNC, #1710), human astrocytes (HA, #1800), human colonic epithelial cells (HCoEpiC, #2950), human cervical epithelium cells (HCerEpic, #7060), human urothelial cells (HUC, #4320) and human neurons (HN, #1520) were purchased from ScienCell/Provitro (Berlin, Germany).The retroviral producer cell line 293vec-Galv were obtained from BioVec Pharma (Quebec, Canada). The retroviral producer cell line PlatE were obtained from Cell Biolabs (San Diego, CA, USA). The mouse cell line 16.113 were obtained from Prof. Dr. Gerald Willimsky (Charité - University Medicine Berlin, Germany) and German Cancer Research Center (DKFZ, Heidelberg, Germany).
Authentication	Cell lines directly and recently (less than 3 years) purchased from internationally recognized vendors were not authenticated. Upon receipt, they were expanded and multiple ampoules were frozen. REH was verified by multiplex cell line authentication test (Multiplexion, Heidelberg, Germany).
Mycoplasma contamination	Cell lines were regularly tested for mycoplasma using MycoAlert assay (Lonza). Cell lines used for the experiments tested negative.
Commonly misidentified lines (See ICLAC register)	Cell lines used for the experiments are not listed in the ICLAC register.

Animals and other organisms

Policy information about studies involving animals; ARRIVE guidelines recommended for reporting animal research

Laboratory animals	For the human CAR experiments, female and male NSG mice (NOD.Cg-Prkdcscid Il2rg tm1 Wjl/SzJ) mice were used (8-12 weeks-old). For the mouse CAR experiments, mixed female and male C57BL/6 mice groups were used as recipients and female mice were used as donors (10-14 weeks old). Female Eμ-Tcl1, and Cxcr5-/-Eμ-Tcl1 transgenic mice (6-10 month old) on a C57BL/6 background were used to harvest tumor cells.
Wild animals	The study did not involve wild animals.
Field-collected samples	The study did not include samples collected from the field.
Ethics oversight	All animal studies were performed according to institutional and Berlin State guidelines of the Landesamt für Gesundheit und Soziales (LAGeSo) (G0373/13; G0050/16; G0104/16).

Note that full information on the approval of the study protocol must also be provided in the manuscript.

Human research participants

Policy information about studies involving human research participants

Population characteristics	Primary B-NHL patient derived material was pseudonymized.
Recruitment	Pre-diagnosed B-NHL patients archived material form tissue bank Charite University Berlin.
Ethics oversight	The study was conducted according to the Declaration of Helsinki and in accordance with local ethical guidelines; written informed consent of all patients was obtained. Usage of primary tumor samples and PBMCs in this study was approved by Ethikausschuss 1 am Campus Charité - Mitte (EA1/003/17; EA1/222/13; EA1/207/14) and Ethikausschuss am Campus Virchow-Klinikum (EA2/216/18). B-NHL subtypes were diagnosed by expert Charité-Universitätsmedizin Berlin hematologists and pathologist.

Note that full information on the approval of the study protocol must also be provided in the manuscript.

Flow Cytometry

Plots

Confirm that:

- [x] The axis labels state the marker and fluorochrome used (e.g. CD4-FITC).
- [x] The axis scales are clearly visible. Include numbers along axes only for bottom left plot of group (a 'group' is an analysis of identical markers).
- [x] All plots are contour plots with outliers or pseudocolor plots.
- [x] A numerical value for number of cells or percentage (with statistics) is provided.

Methodology

Sample preparation	All reagents and mAbs for flow cytometric analysis were purchased from Biolegend/Biozol unless stated otherwise. FcR block was performed for 15 min at RT with either 5% AB serum (Sigma-Aldrich), human or mouse TruStein FcX in FACS buffer (PBS, 2% FCS, 10 mM EDTA); this reagent was omitted if an anti-IgG Ab was used. Where appropriate, True-Stain Monocyte Blocker was included. Antibody were conducted for 15 min at RT in the dark. Cells were subsequently washed three times and resuspended in FACS buffer. Dead cells were excluded by staining with 7-AAD. Zombi AquaDye (ThermoFisher Scientific). Specific staining of the mAbs was verified by isotype control staining.
Instrument	FACS Canto II (BD), Aurora Spectral Cytometer (Cytek)
Software	FACS Diva version 6.1.3 (BD), SpectroFlo version 2.2.0 (Cytek)
Cell population abundance	Purity of sorted cells was analyzed by flow cytometry.
Gating strategy	FSC-H/FSC-W -> singlets, SSC-H/SSC-W -> singlets, FSC-A/SSC-A -> lymphocytes, SSC-A/7-AAD -> live cells

- [x] Tick this box to confirm that a figure exemplifying the gating strategy is provided in the Supplementary Information.
